# The Study of Antistaphylococcal Potential of Omiganan and Retro-Omiganan Under Flow Conditions

**DOI:** 10.1007/s12602-023-10197-w

**Published:** 2024-01-15

**Authors:** Maciej Jaśkiewicz, Damian Neubauer, Karol Sikora, Marta Bauer, Sylwia Bartoszewska, Izabela Błażewicz, Dariusz Marek, Wioletta Barańska-Rybak, Wojciech Kamysz

**Affiliations:** 1 Department of Inorganic Chemistry, Faculty of Pharmacy, Medical University of Gdańsk, Al. Gen. J. Hallera 107, 80-416 Gdańsk, Poland; 2 International Research Agenda 3P-Medicine Laboratory, Medical University of Gdańsk, Building No. 5, Dębinki 7, 80-211 Gdańsk, Poland; 3 Department of Dermatology, Venereology and Allergology, Medical University of Gdańsk, Mariana Smoluchowskiego 17, 80-214 Gdańsk, Poland

**Keywords:** Antimicrobial peptides, Biofilm, Counterions, *Staphylococcus aureus*, Omiganan, PDMS

## Abstract

*Staphylococcus aureus* is considered one of the leading pathogens responsible for infections in humans and animals. The heterogeneous nature of diseases caused by these bacteria is due to the occurrence of multiple strains, differentiated by several mechanisms of antibiotic resistance and virulence factors. One of these is the ability to form biofilm. Biofilm-associated bacteria exhibit a different phenotype that protects them from external factors such as the activity of immune system or antimicrobial substances. Moreover, it has been shown that the majority of persistent and recurrent infections are associated with the presence of the biofilm. Omiganan, an analog of indolicidin - antimicrobial peptide (AMP) derived from bovine neutrophil granules, was found to exhibit high antistaphylococcal and antibiofilm potential. Furthermore, its analog with a reversed sequence (retro-omiganan) was found to display enhanced activity against a variety of pathogens. Based on experience of our group, we found out that counterion exchange can improve the antistaphylococcal activity of AMPs. The aim of this study was to investigate the activity of both compounds against *S. aureus* biofilm under flow conditions. The advantage of this approach was that it offered the opportunity to form and characterize the biofilm under more controlled conditions. To do this, unique flow cells made of polydimethylsiloxane (PDMS) were developed. The activity against pre-formed biofilm as well as AMPs-treated bacteria was measured. Also, the incorporation of omiganan and retro-omiganan into the channels was conducted to learn whether or not it would inhibit the development of biofilm. The results of the microbiological tests ultimately confirmed the high potential of the omiganan and its retro-analog as well as the importance of counterion exchange in terms of antimicrobial examination. We found out that retro-omiganan trifluoroacetate had the highest biofilm inhibitory properties, however, acetates of both compounds exhibited the highest activity against planktonic and biofilm cultures. Moreover, the developed methodology of investigation under flow conditions allows the implementation of the studies under flow conditions to other compounds.

**Supplementary Information:**

The online version contains supplementary material available at 10.1007/s12602-023-10197-w.

## Introduction

*Staphylococcus aureus* is one of the dominant pathogens responsible for infections of animals and humans. These infections can be characterized by different localization and severity and can be caused by different strains among this group. Nevertheless, humans are natural reservoirs for these bacteria with a suggestion that up to 50% of healthy adults are colonized [1] and persistent nasal carriage is indicated as the dominating one [2]. Another characteristic fact is that *S. aureus* can easily be transferred between healthy human carriers and animals. This leads to the occurrence of the multiplicity of strains, characterized by a variety of virulence factors, which are crucial for adaptation, survival, and spread [3–5]. Multidrug resistance and the ability to form biofilm are the key virulence factors that are associated with prolonged and insufficient therapy. For instance, methicillin-resistant strains (MRSA) are those that are responsible for the significant amount of hospital-acquired infections (HAIs) and overall costs of healthcare globally [6–10]. The most common infections caused by *S. aureus* are skin and soft tissue infections (SSTIs) and bacteremia. However, infective endocarditis, osteomyelitis, and prosthetic joint infections overwhelmingly involve *S. aureus* as a primary etiological factor [11–18, 21–25]. In view of the soaring resistance to antibiotics, the search for new effective antimicrobial compounds is still highly demanded, and antimicrobial peptides (AMPs) stand out as a promising solution to address this challenge. AMPs are compounds widespread in nature and can be found in almost all kingdoms of organisms in which they act as a part of innate immunity [19]. A characteristic feature of these compounds is a broad spectrum of antimicrobial activity, which covers bacteria, fungi, protozoa, and viruses [20]. Moreover, endogenous AMPs can trigger innate immunity and, unlike conventional antibiotics, they are characterized by a high activity against biofilm [21]. Undoubtedly, the synthetic approaches allowed to obtain peptides with biological activity based on their natural structure, but due to the presence of amino acids, their application in therapy is still limited. For instance, several obstacles need to be considered, such as toxicity, stability, solubility, and pharmacokinetic profile of final formulations. In our group, we have introduced several strategies to overcome those limitations and to enhance their antistaphylococcal activity. Among these, the synthesis of analogs with reversed sequence (retro-analogs) as well as counterion exchange can be highlighted as promising approaches for designing AMPs. In our works, we have proven that for some peptides reversion of the sequence caused significant improvement in antimicrobial activity while the kind of counterions may be crucial for antistaphylococcal activity and cytotoxicity as well [22, 23]. Omiganan (MBI-226) is one of the most studied synthetic AMPs for which the largest number of phase III clinical trials has been completed [24]. For instance, it was demonstrated to be effective in the reduction of catheter microbial colonization. However, most studies were focused on topical gel evaluation in the treatment of *S. aureus*-associated infections such as acne vulgaris, atopic dermatitis, and rosacea [25]. For its wide spectrum of antimicrobial activity, omiganan was chosen for several studies in our group. Interestingly, its retro-analog appeared to be one of the AMPs tested for which the reversion of the sequence led to enhancement of the antimicrobial activity against bacteria and fungi as well as suppressed hemolytic potential [22]. In further studies, it was found that retro-omiganan exhibits a higher activity against the *Acinetobacter baumannii* [26] and *Candida albicans* strains isolated from vulvovaginal candidiasis than that of the parent molecule. Based on these reports and our previous works focused on antistaphylococcal activity of synthetic AMPs [27–30], we decided to initiate a comprehensive study on the activity of omiganan and retro-omiganan that combines chemical and microbiological approaches. In this study, for both peptides, counterion exchange was conducted and verified in terms of the activity against *S. aureus* reference and clinical strains. These experiments included minimum inhibitory concentration (MIC) determination and impact on biofilm in terms of minimum biofilm inhibitory concentration (MBIC) and minimum biofilm eradication concentration (MBEC). To verify the selectivity of these compounds, the % hemolysis against human red blood cells (hRBCs) and half-maximal inhibitory concentration (IC_50_) against HaCaT cell line were evaluated. Moreover, membrane permeabilization characteristics were examined. Finally, to confirm the potential of using those AMPs in the prevention of catheter-associated infections, we designed unique flow cell chambers made of polydimethylsiloxane (PDMS) to investigate the activity of omiganan and retro-omiganan under flow conditions. Since the growth of biofilm under dynamic conditions was found to mimic environmental conditions and those encountered in vivo, it was reasonable to conduct such an examination [31]. For this purpose, the activity against pre-formed biofilm as well as AMPs-treated bacteria was measured. In addition, the incorporation of omiganan and retro-omiganan into the channels was conducted to learn whether or not it would inhibit the development of biofilm. The aim of the study was to thoroughly determine the activity of the tested peptides against staphylococci. The choice of an adequate counterion is of application importance, as it determines not only the microbial activity but also the toxicity and thus the final pharmaceutical formulation. Subsequently, the peptide content was measured to indicate more precisely the activity of test compounds. The utilization of clinical strains previously examined by our group for antibiotic susceptibility and the presence of resistance genes enabled us to select the most interesting strains. These particular ones were isolated from skin and nasal swabs from patients with atopic dermatitis [29]. The selection of *S. aureus* as the research model is not accidental, because these bacteria are the leading cause of nosocomial and catheter-related infections (CRIs), while the development of a unique biofilm flow model allowed for even more in-depth understanding of the activity of the test compounds and their potential use in prevention of catheter-related infections. According to our knowledge, there has not been a comprehensive, wide-raging study on AMPs conducted by any scientific group so far. Moreover, our approach could give guidance to other groups on how research on synthetic peptides can be arranged. It could give insights into the application of some compounds that do not meet the cytotoxicity criteria but could be applied for biomaterials functionalization [32]. Nevertheless, some limitations of the study should be emphasized. For instance, the chosen counterions can act differently on other cell lines. Moreover, infections encountered in vivo may be caused by multiple microorganisms and the biofilm can be formed differently on other biomaterials.

## Materials and Methods

### Peptide Synthesis

Omiganan, retro-omiganan, and melittin were synthesized manually by solid-phase peptide synthesis (SPPS) method using Fmoc chemistry on polystyrene resin modified by a Rink amide linker (4-(2′,4′-dimethoxyphenyl-Fmoc-aminomethyl)-phenoxymethyl resin, particle size 100–200 mesh, loading 0.67 mmol/g, crosslinking degree 1% divinylbenzene; Sunresin, Xi’an, Shaanxi, China). Deprotection of the Fmoc groups was carried out in a 20% (v/v) piperidine (Merck, Darmstadt, Germany) solution in DMF (*N*,*N*-dimethylformamide; Honeywell, Seelze, Germany) with constant shaking for 15 min at room temperature. Attachment of protected amino acids was conducted in a DMF/DCM solution (1:1, v/v, DCM—dichloromethane; Chempur, Piekary Śląskie, Poland) with coupling agents using a threefold molar excess of DIC (*N*,*N*′-diisopropylcarbodiimide; Peptideweb, Zblewo, Poland) and OxymaPure (Iris Biotech GmbH, Marktredwitz, Germany) with constant shaking for 1.5 h at room temperature. Therefore, acylation was conducted with a mixture of DIC:OxymaPure:Fmoc-AA-OH (molar ratio 1:1:1) at concentration of 0.1 M. *N*^*α*^-Fmoc-protected amino acids were obtained from Carbolution Chemicals GmbH (St. Ingbert, Germany). The following amino acids side-chain-protecting groups were used: Trt—trityl (for Gln), tBu—*tert*-butyl (Ser and Thr), Boc—*tert*-butoxycarbonyl (Lys and Trp), Pbf—2,2,4,6,7-pentamethyldihydrobenzofuran-5-sulfonyl (Arg). All reactions were performed using a Kamush peptide shaker (Kamush, Poland). Every step was preceded by rinsing the resin with DMF (3 ×) and DCM (3 ×). Chloranil test was used to control acylation and deprotection processes. Briefly, a few mg of resin were placed in a small test tube. Next, 1 drop of 2% acetaldehyde (Sigma-Aldrich Chemie GmbH, Buchs, Switzerland) in DMF and 1 drop of 2% p-chloranil (Merck KGaA, Darmstadt, Germany) in DMF were added and incubated at room temperature for 5 min. If the beads were blue, free amine groups were present and the coupling reaction was not complete. In such case, second coupling was performed. If the beads remained unstained, coupling reaction was completed. After the synthesis, the peptide resins were dried under vacuum. The peptides were cleaved from the resin using the mixture of TFA (trifluoroacetic acid; Apollo Scientific, Denton, UK), phenol (Sigma-Aldrich, St. Louise, MO, USA), triisopropylsilane (TIS) (Sigma-Aldrich, St. Louise, MO, USA), and deionized water (92.5:2.5:2.5:2.5 v/v). Cleavage from the resin was accomplished for 1.5 h with agitation. Crude peptides were precipitated with cold diethyl ether (Chempur, Piekary Śląskie, Poland) and centrifuged (3461 × g, 5 min; EBA 20, Hettich, Andreas Hettich GmbH & Co. KG, Tuttlingen, Germany). The supernatant was discarded, and the crude peptide was dissolved in deionized water and lyophilized. Purification of the compounds was carried out by RP-HPLC on a Phenomenex Gemini-NX C18 column (21.20 × 100 mm, 5.0 μm particle size, 110 Å pore size) with UV detection at 214 nm. Eluents used were deionized water and acetonitrile (Fisher Scientific, Belgium) containing 0.1% (v/v) of TFA. A linear 10–70% acetonitrile gradient in deionized water over 90 min was used and mobile phase flow rate was 20.0 mL/min. The purity and identity of the peptides was confirmed with LC–MS analysis. The RP-HPLC system was used—Waters Alliance e2695 system with Waters 2998 PDA and Acquity QDA detectors (software—Empower®3, Waters, Milford, MA, USA). All analyses were carried out on a Waters XBridge™ Shield RP-18 column (3.0 × 100 mm, 3.5 µm particle size, 130 Å pore size). Samples (10 µL) were analyzed with a linear 10–90% acetonitrile gradient in deionized water over 15 min at 25.0 ± 0.1 °C. The mobile phase flow rate was 0.5 mL/min. Both eluents contained 0.1% (v/v) of formic acid (Sigma-Aldrich Chemie GmbH, Steinheim, Germany). Mass analysis and UV detection at 214 nm were used. Pure fractions (> 95%, by HPLC analysis) were collected and lyophilized (Table 1).

**Table 1:** Peptides used in this study

**Name**	**Sequence**	**Average mass (Da)**	**Net charge**	**MS analysis**		
				**z**^**a**^	**m/z**^**b**^	**m/z**^**c**^
Melittin	GIGAVLKVLTTGLPALISWIKRKRQQ-NH_2_	2846.46	+6	3	950.1	949.8
				4	712.8	712.9
				5	570.4	570.5
Omiganan	ILRWPWWPWRRK-NH_2_	1779.15	+5	2	890.6	890.3
				3	594.1	594.1
				4	445.8	445.9
Retro-omiganan	KRRWPWWPWRLI-NH_2_	1779.15	+5	2	890.6	890.3
				3	594.1	594.1
				4	445.8	446.0

^a^Ion charge

^b^Calculated mass to charge ratio

^c^Measured mass to charge ratio

### Counterion Exchange and Ion Chromatography

Omiganan and retro-omiganan were obtained initially as TFA salts. Counterions were then exchanged to biocompatible acetates (AcO^−^) and hydrochlorides (Cl^−^). The exchange to AcO^−^ was accomplished in two steps. First, TFA anions were removed with a carbonate ion-exchange resin. To achieve that, the peptide was dissolved in water and passed through commercially available ion-exchange columns—VariPure (Agilent). The resin was washed with water and acetonitrile; fractions were combined, and acetonitrile was removed under reduced pressure in the rotary evaporator. Subsequently, acetic acid was added to the peptide solution, and the samples were lyophilized. The exchange to Cl^−^ was performed using HCl-saturated acetonitrile as reported previously [33]. Briefly, the peptide was dissolved in acetonitrile saturated with HCl (0.5%), incubated at room temperature for 15 min, and evaporated to dryness in rotary evaporator at 40 °C. Whole process was repeated twice. After that peptide sample was dissolved in water and lyophilized to remove excess of HCl. All samples were analyzed by ion chromatography (IC) (Dionex ICS-5000+, Thermo-Scientific). The method was validated for the analysis of TFA^−^, AcO^−^, and Cl^−^ according to the ICH guidelines Q2 (R1) [34]. The analyses were performed with isocratic elution (4.5 mM Na_2_CO_3_ and 1.4 mM NaHCO_3_ in water), a flow rate of 1.2 mL/min, and an injection volume of 20 µL. All the tested samples were dissolved in water up to a concentration of 0.5 mg/mL. Ions were detected by a conductivity detector coupled with ASRS 300—anion self-regenerating suppressor and the suppressor current of 31 mA. Dionex IonPac AS22 dimensions 4.0 × 250 mm column was used. Column compartment temperature was set at 30 ± 0.1 °C and conductivity detector temperature was 35 ± 0.1 °C.

### Measurement of Peptide Content

Peptide concentration was estimated spectrophotometrically by absorbance measurements at 280 nm (Multiskan™ GO Microplate Spectrophotometer, Thermo Scientific) based on the presence of tryptophan (*W*) and tyrosine (*Y*) residues in the sequence. For this purpose, increasing concentrations of the peptide salts were used, namely, 0.0125, 0.025, 0.050, 0.075, 0.1, 0.125, and 0.25 mg/mL, to ensure that the measurements were accomplished within the linearity range. All samples were dissolved in a 6 M guanidine hydrochloride, pH 6.5, 0.02 M phosphate buffer and the measurements were conducted in a quartz cuvette with a 10 mm path length. Since the molar extinction coefficient (*ɛ*) is constant and additive for *W* (5560 AU/mmol/mL) and *Y* (1200 AU/mmol/mL), the peptide concentration was calculated from the following equation [35, 36]:

$$\mathbf{P}\mathbf{e}\mathbf{p}\mathbf{t}\mathbf{i}\mathbf{d}\mathbf{e}\mathbf{c}\mathbf{o}\mathbf{n}\mathbf{c}\mathbf{e}\mathbf{n}\mathbf{t}\mathbf{r}\mathbf{a}\mathbf{t}\mathbf{i}\mathbf{o}\mathbf{n}[\mathbf{m}\mathbf{g}/\mathbf{m}\mathbf{L}]=\frac{({\varvec{A}}\times {\varvec{D}}{\varvec{F}}\times {\varvec{M}}{\varvec{W}})}{[\left({\varvec{W}}\times{\varvec{\varepsilon}}\right)+\left({\varvec{Y}}\times{\varvec{\varepsilon}}\right)]}$$

where *A*—absorption at 280 nm [AU]; *DF*—dilution factor; *MW*—molecular mass [mg × mmol^−1^]; *W*—number of tryptophan residues; *Y*—number of tyrosine residues; *ɛ*—molar extinction coefficient at 280 nm [cm^−1^ × M^−1^].

### Antimicrobial Assays

Clinical *S. aureus* strains were collected from patients with atopic dermatitis during their visits to the Outpatient Clinic and hospitalization in the Department of Dermatology, Venereology and Allergology at Medical University of Gdańsk (following the approval from the ethics committee, Approval Number NKBBN/242–477/2014). Reference strains of *S. aureus* ATCC 25923, *S. aureus* ATCC 33591, *S. aureus* ATCC 9144, and *S. aureus* ATCC 12598 were obtained from the American Type Culture Collection. All the strains were stored at − 80 °C in Roti®-Store cryo vials (Carl Roth GmbH, Karlsruhe, Germany) and before the tests were transferred into fresh Mueller–Hinton Medium (BioMaxima, Lublin, Poland) and incubated for 24 h at 37 °C. Subsequently, each bacterial inoculum was seeded on Mueller–Hinton agar plates (BioMaxima) and incubated again for 24 h. The cultures prepared in this way were used in further antimicrobial assays. Minimum inhibitory concentrations (MICs) were determined according to the Clinical and Laboratory Standards Institute recommendations [37]. Briefly, the initial inoculums of bacteria (0.5 × 10^5^ CFU/mL) in the Mueller–Hinton broth (MHB) were exposed to the ranging concentrations of peptides (0.5–256 μg/mL) and incubated for 18 h at 37 °C. The experiments were conducted on 96-well microtiter plates, with a final volume of 100 μL. Additionally, in the case of daptomycin, the medium was supplemented with Ca^2+^ (50 mg/L). Cell densities were adjusted spectrophotometrically (Multiskan™ GO Microplate Spectrophotometer, Thermo Scientific) at 600 nm. The MICs were taken as the lowest drug concentration at which a visible growth of the microorganisms was inhibited. Minimum biofilm eliminating concentrations (MBECs) were determined on 96-well flat-bottomed microtiter polystyrene plates with resazurin (7-hydroxy-3H-phenoxazin-3-one 10-oxide sodium salt) as a cell viability reagent. To do this, the plates were filled with 100 μL of the initial inoculums of bacteria (0.5 × 10^7^ CFU/mL) in the Mueller–Hinton broth and incubated for 24 h at 37 °C. Subsequently, the wells were rinsed three times with PBS to remove non-adhered cells and the fresh medium (100 μL) with a series of concentrations (0.5–256 μg/mL) of the peptides was added. After 24 h of incubation, 20 μL of resazurin (4 mg/mL) was added to each well and the MBEC values were read. Minimum biofilm inhibitory concentrations (MBICs) were also determined on 96-well flat-bottomed microtiter polystyrene plates as described previously [38]. To do this, 50 μL of the compounds in the concentration range, diluted MHB, were prepared. Subsequently, the 50 μL of the staphylococcal inoculums were added to reach the same density of bacteria as that in the MBEC assay. After 24 h of incubation at 37 °C, the wells were rinsed three times with PBS and the fresh medium (100 μL) with resazurin (0.6 mg/mL) was added. Then, after 1 h of incubation, MBICs were read. MBECs as well as MBICs were determined as the lowest concentration at which the reduction of resazurin was lower or equal (10% ± 0.5%) as compared to either the positive (100%) or negative (0%) controls. All experiments were conducted in triplicate.

### MTT Assay

To evaluate the cytotoxicity of the peptides (IC_50_), the classic MTT assay on 96-well plates was performed for human keratinocytes (HaCaT) which were acquired from the ATCC. The assay utilizes colorimetric determination of the cell metabolic activity, and the color intensity reflects the number of live cells that can be measured spectrophotometrically. The cell line was cultured in the Dulbecco’s modified Eagle Medium (Invitrogen) supplemented with a 10% fetal bovine serum (v/v), 100 units/mL of penicillin, 100 μg/mL of streptomycin, and 2 mM l-glutamine and was kept at 37 °C in a humidified 5% CO_2_ incubator. Briefly, a day after plating of 500 cells per well, a series of concentrations (0.5–500 μg/mL) of the test compounds were applied. DMSO was added to the control cells at a final concentration of 1.0% (v/v), which was related to the maximum concentration of the solvent compounds used in the experiment. After 24 h of incubation at 37 °C (humidified 5% CO_2_ incubator) with the peptides, a medium containing 1 mg/mL of MTT was added to the wells up to a final concentration of 0.5 mg/mL. Subsequently, the plates were incubated at 37 °C for 4 h. Then, the medium was aspirated, and the formazan product was solubilized with DMSO. The background absorbance at 630 nm was subtracted from that at 570 nm for each well (Epoch, BioTek Instruments, USA). Six replicates were conducted for each concentration. All experiments were repeated at least twice and the resulting IC_50_ values were calculated with GraFit 7 software (v. 7.0, Erithacus, Berkley, CA, USA).

### Hemolytic Activity

The hemolysis assay was conducted using a procedure described previously [39]. For this purpose, fresh human red blood cells (RBCs) with EDTA as anticoagulant were washed three times with phosphate-buffer saline (PBS) by centrifugation at 800 × g for 10 min and resuspended in PBS. Then, the stock solution of RBCs was added to serial dilution of peptides on 96-well polystyrene plates to reach a final volume of 100 µL with 4% concentration of erythrocytes (v/v) and a concentration range of 0.5–256 µg/mL of tested compounds. The control wells for 0% hemolysis and 100% hemolysis consisted of RBCs suspended in PBS and 1% of Triton X–100, respectively. Subsequently, the plates were incubated for 60 min at 37 °C and then centrifuged at 800 × g for 10 min at 4 °C (Sorvall ST 16R Centrifuge, Thermo Scientific). After centrifugation, the supernatant was carefully resuspended to new microtiter plates and the release of hemoglobin was monitored by absorbance readings at 540 nm (Multiskan™ GO Microplate Spectrophotometer, Thermo Scientific). All experiments were conducted in triplicate. Blood collection was approved by Medical University of Gdańsk ethics committee (Consent Number: NKBBN/264/2019).

### Membrane Permeabilization Assay

Membrane depolarization activities of omiganan and retro-omiganan were evaluated using DiSC_3_(5) (3,3′-dipropylthiadicarbocyanine iodide; Thermo Fischer Scientific, Invitrogen™). The *S. aureus* ATCC 25923 and *S. aureus* ATCC 33591 strains were grown at 37 °C up to a mid-log phase (approx. 4 h) in the Mueller–Hinton broth. The cultures were centrifuged (3500 rpm, 7 min) and washed with a 20 mM glucose solution in HEPES buffer (5 mM, pH 7.2). The cells were resuspended in a 5 mM HEPES buffer supplemented with 20 mM glucose and 100 mM KCl (pH 7.2) to an OD 0.05 at 600 nm. The final concentration of the dye was 0.4 μM. The fluorescence was monitored at 20 °C (λ_ex_ 620 nm and λ_em_ 678 nm) with Fluoroskan Ascent FL (Thermo Fisher Scientific) fluorometer. As soon as the dye uptake attained a maximum, the peptides were added at a concentration of 2 × MIC. Melittin is known as an effective membrane disruptor, and as such it was used as a positive control (2 × MIC; 64 μg/mL). A HEPES solution with glucose was used as a negative control. The measurements were conducted twice to ensure the reproducibility.

### Preparation of PDMS Flow Chambers

**Fig. 1:**
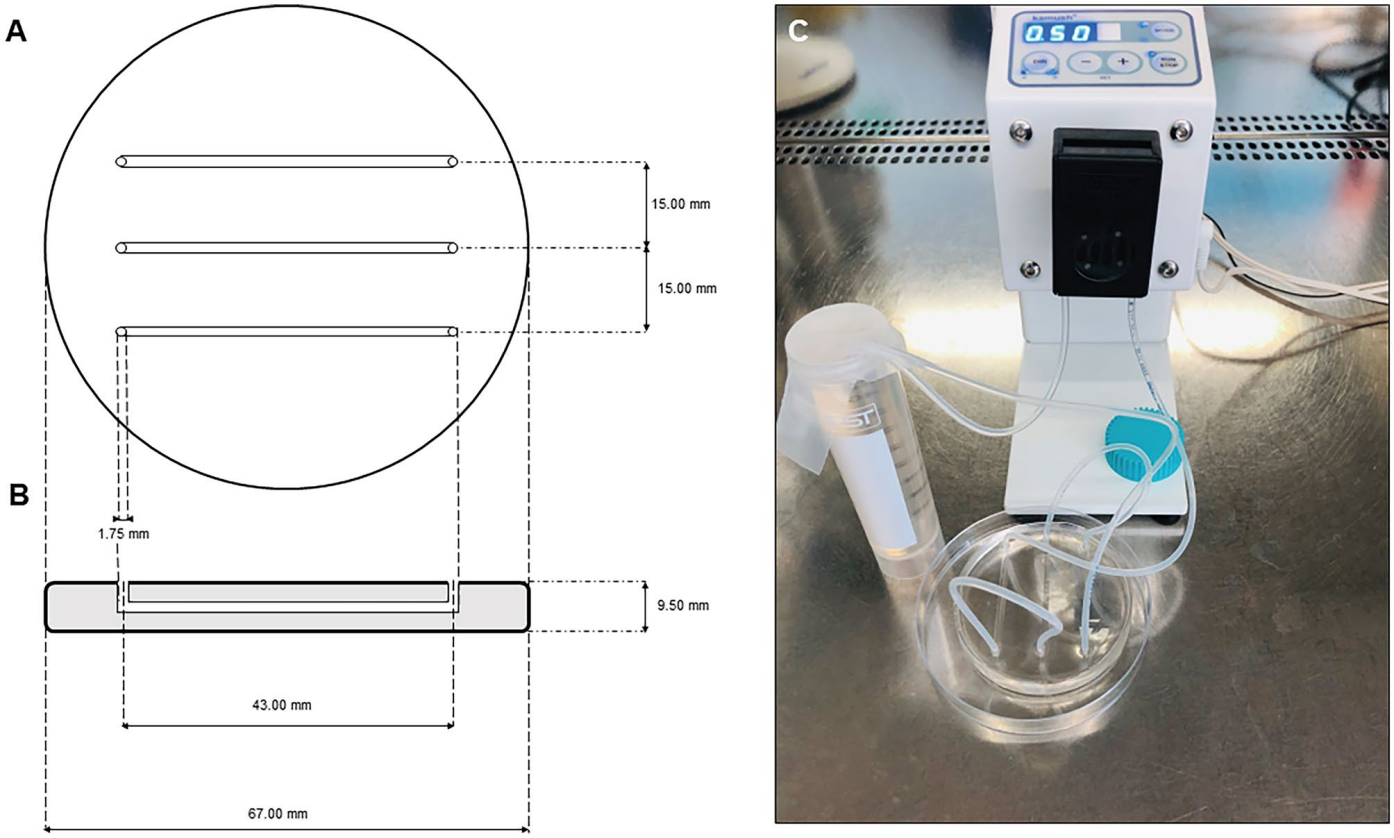
The diagram of PDMS flow chambers. **A** Front view; **B** side view. **C** Photograph of the pre-prepared system

The PDMS flow chambers were prepared using a two-component Sylgard 184 kit (Dow Corning, MI, USA) according to the manufacturer’s instructions. The base and the curing solution were mixed in a ratio at 10:1 (m/m) and poured onto glass Petri dishes. Subsequently, the racks with ABS (acrylonitrile butadiene templates) were immersed in solution (the shape and dimensions of the systems are shown in Fig. 1) and left for cross-linking at room temperature for 48 h. All channels were designed to obtain a final volume of 100 µL, whereas their internal diameters corresponded to those found in typical intravascular catheters [40]. Then, the ABS templates were removed to form channels in PDMS. This was achieved by dissolving ABS with acetone in the ultrasonic bath. PDMS flow chambers were washed with methanol, dried (5 min), and autoclaved in sterilization sleeves for 15 min at 121 °C.

### Biofilm Formation Under Flow Conditions

**Fig. 2:**
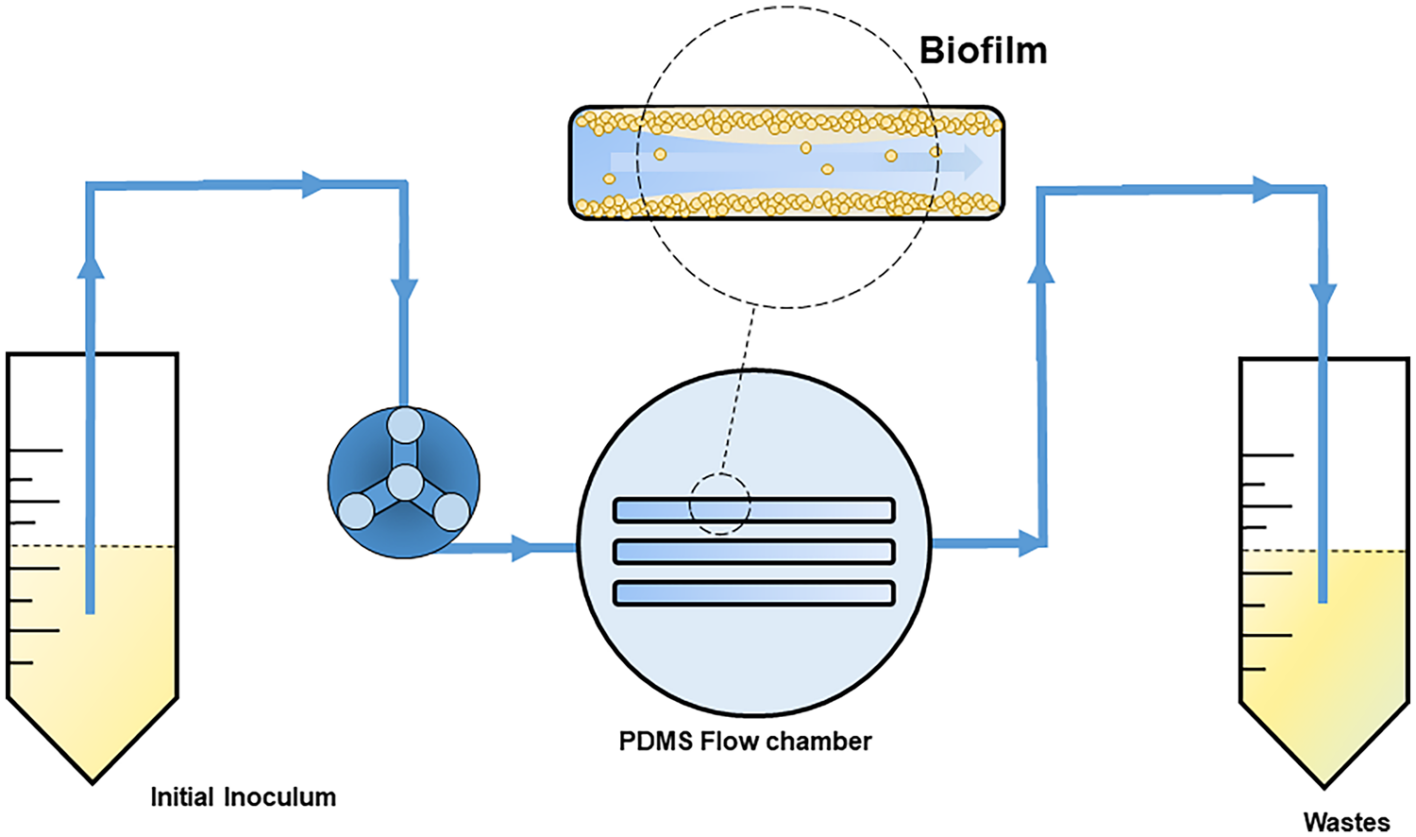
The schematic presentation of the PDMS-based biofilm flow system

Formation of the biofilm inside the PDMS flow chambers was performed using three peristaltic pumps working simultaneously (LP-pump, Kamush, Lipopharm, Poland) and sterile Tygon® 3350 silicone hoses (2.4 × 0.8 × 0.8 mm) with a length of 30 and 50 cm for inlet and outlet, respectively. Bacterial suspensions were prepared (0.5 × 10^7^ CFU/mL) in sterile 50-mL test tubes, which were placed in a heating block at 37 °C. Before experiments, all channels were assessed for tightness and sterilized using a 70% 2-propanol solution (10 min, flow rate 1 mL/min) and rinsed with sterile PBS solution (10 min, flow 0.1 mL/min). The formation of biofilm inside the channels was divided into three stages: (1) adhesion—at this stage *S. aureus* suspensions were passed through the channels for 4 h at a flow rate of 0.1 mL/min; (2) rinsing—the previously used hoses were replaced with new sterile ones, and the channels were rinsed with sterile PBS solution for 10 min at a flow rate of 0.1 mL/min; (3) biofilm growth—after flushing the channels, the silicone hoses were placed in tubes with MHB medium, which was passed through the channels for 24 h at a flow rate of 0.1 mL/min. All experiments included growth and sterility controls. A schematic presentation of the used system is shown in Fig. 2.

### Biofilm Eradication Under Flow Conditions

This assay was conducted on model *Staphylococcus* reference strains: *S. aureus* ATCC 25923 and *S. aureus* 33,591 (MRSA). To do this, the biofilm was formed inside the PDMS channels as described above. After biofilm formation stage, the channels were rinsed with a sterile PBS solution (10 min, 0.1 mL/min) to remove non-adherent cells. Then, the medium supplemented with a test compound was pumped through the channel for 24 h (0.1 mL/min). Then, the content of the channels was rinsed with 10 mL of PBS in a closed circuit under a high flow rate (10 min, 15 mL/min) to detach the biofilm-associated bacteria. The completeness of detachment was confirmed by crystal violet staining and microscopic inspection. One hundred microliters of each suspension was transferred into a 96-well flat-bottom plate, 20 μL of resazurin (4 mg/mL) was added to each well, and the MBEC values were read.

### Susceptibility Profile of *S. aureus* Exposed to the Peptides Under Flow Conditions

This assay was conducted also on the *S. aureus* ATCC 25923 and *S. aureus* 33,591 (MRSA) strains. The biofilm was formed inside the PDMS channels as described above. After biofilm formation, the channels were rinsed with a sterile PBS solution (10 min, 0.1 mL/min) to remove non-adherent cells. Then, the medium supplemented with the test compound at a concentration equal to half of the MBEC value was pumped through the channel for 24, 48, and 72 h, respectively (flow rate 0.1 mL/ min). After each exposure step, each channel was rinsed with a sterile PBS solution (10 min, 0.1 mL/min). Then, the content of the channels was rinsed with 10 mL of PBS in a closed circuit under a high flow (10 min, 15 mL/min) to detach the biofilm-associated bacteria. The completeness of detachment was confirmed by crystal violet staining and microscopic inspection. The resulting suspension was then inoculated onto MHA which was incubated for 24 h at 37 °C. In this way, different *S. aureus* clones were obtained which were treated with the tested peptide for 24, 48, and 72 h. These strains were further used for MIC determination. All experiments were conducted in triplicate.

### Impact of Co-immobilized Peptides on Biofilm Formation Under Flow

**Fig. 3:**
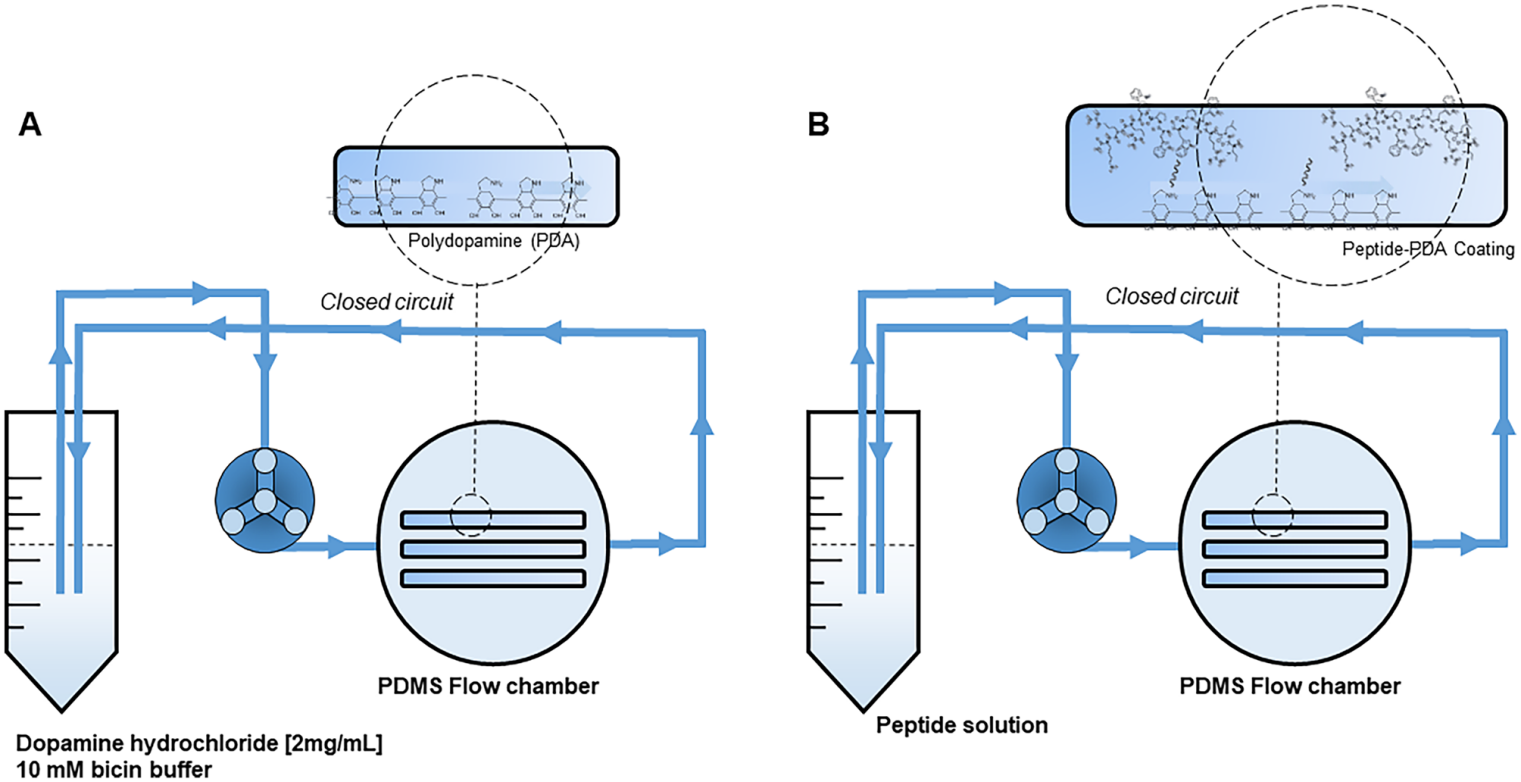
Schematic presentation of immobilization of the peptides into PDMS chambers. **A** First step: preparation of pDa coating. **B** Co-immobilization of the peptides

Incorporation of omiganan and retro-omiganan into PDMS channels was conducted using a polydopamine (pDa) coating, as previously described with a slight modification resulting from flow-based experimental model [41]. To do this, the pDa coating was done by rinsing the channels with a solution of dopamine hydrochloride (Sigma-Aldrich, 2 mg/mL) in 10 mM bicin buffer (pH 8.5) for 18 h, 0.3 mL/min in a closed system (Fig. 3A). Then, the channels were rinsed with a sterile solution of demineralized water. Further co-immobilization was conducted using new, sterile silicone hoses connected to flow chambers and test tubes containing aqueous solutions of the peptides (100 µg/mL) pumped through the channels in a closed system for 6 h at 0.3 mL/min (Fig. 3B). Assessment of peptide binding was performed using liquid chromatography (RP-HPLC), by comparing the content of the compounds in the solution before and after incorporation. Waters Alliance e2695 system with a Waters 2998 PDA Detector (software-Empower 3, Waters, Milford, MA, USA) was used. All analyses were carried out on a Waters X-Bridge Shield RP-18 column (3.0 × 100 mm, 3.5 μm particle size, 130 Å pore size) with UV detection at 214 nm and samples (10 μL) were eluted with a linear 10–90% acetonitrile gradient in deionized water over 15 min at 25.0 ± 0.1 °C. The mobile phase flow rate was 0.5 mL/min. Both eluents contained 0.1% (v/v) of TFA. Each peptide sample was analyzed in triplicate. Such functionalized PDMS channels were used for further studies on biofilm formation. The formation of the biofilm followed the procedure described above and was conducted for *S. aureus* ATCC 25923 and *S. aureus* ATCC 33591 (MRSA). After 24 h, the channels were rinsed with 10 mL of PBS in the closed circuit (10 min, 15 mL/min) to detach the bacteria. The obtained bacterial solutions were then diluted and inoculated on MHA plates to count the number of bacteria forming the biofilm inside the channels. The % reduction of staphylococci was estimated by the ratio of bacteria from the immobilized and non-immobilized peptide channels. All experiments were conducted in triplicate.

## Results

### Peptide Synthesis

#### Counterion Exchange

For this study, antimicrobial characteristics of three salts were evaluated, namely, acetates, chlorides, and trifluoroacetates. Since SPPS peptides are obtained as TFA salts, the exchange into acetates and chlorides was performed. The efficiency of counterion exchange was measured by ion chromatography. The content of AcO^−^ was 87.31 and 86.26% µmol for omiganan and retro-omiganan, respectively (Table 2). With the chlorides, the counterion exchange was much more effective and resulted in total amount of Cl^−^ of 97.81 and 100% µmol, respectively. With acetates, the trace amounts of TFA^−^ and Cl^−^ were noticed. The latter was found in a small amount in the columns with ion exchange resin, which in this case are used to eliminate the trifluoroacetate ions. Furthermore, some traces of TFA anions were also spotted for omiganan chloride.

**Table 2:** The quantities of acetate, chloride, and trifluoroacetate anions in the peptides after counterion exchange

**Peptide**	**Counterion**	**% µmol**		
		**TFA**^**−**^	**AcO**^**−**^	**Cl**^**−**^
Omiganan	TFA^−^	100.00	0.00	0.00
Retro-omiganan		100.00	0.00	0.00
Omiganan	AcO^−^	3.41	87.31	9.28
Retro-omiganan		4.28	86.26	9.46
Omiganan	Cl^−^	2.19	0.00	97.81
Retro-omiganan		0.00	0.00	100.00

### Measurement of Peptide Content

In the case of SPPS, the final product is obtained as a lyophilizate, which includes not only the peptide itself, but also counterions and water. Due to the hygroscopic nature of peptide lyophilizates, the amounts of these impurities vary upon storage. Without thorough analyses (e.g., amino acid analysis or the use of absorption of ultraviolet radiation by tryptophan and tyrosine), it is impossible to precisely determine the concentration of a peptide in the sample [42]. This type of analysis is of particular importance in the case of proteomic analyses, when it is necessary to precisely determine the concentration of specific proteins (markers) in samples taken from patients. In the case of the counterion exchange procedure, which is associated with multiple lyophilizations, there is a risk of changing the real content of the peptide in the lyophilizate. Therefore, to accurately examine the impact of the exchange of counterions on the antistaphylococcal activity of omiganan and its retro-analog, the content of the peptides in the lyophilizates was determined. Owing to the presence of 4 tryptophan residues in the sequence (ILRWPWWPWRRK-NH_2_), it was possible to determine the content of the peptide (Table 3).

**Table 3:** Peptide content before and after counterion (TFA^−^) exchange

**Peptide**	**Counterion**	**Peptide content [mg/mg]**
Omiganan	TFA^−^	1.00 ± 0.06
Retro-omiganan		0.96 ± 0.05
Omiganan	AcO^−^	0.85 ± 0.03
Retro-omiganan		0.79 ± 0.02
Omiganan	Cl^−^	0.85 ± 0.03
Retro-omiganan		0.75 ± 0.02

### Antimicrobial Assays

**Fig. 4:**
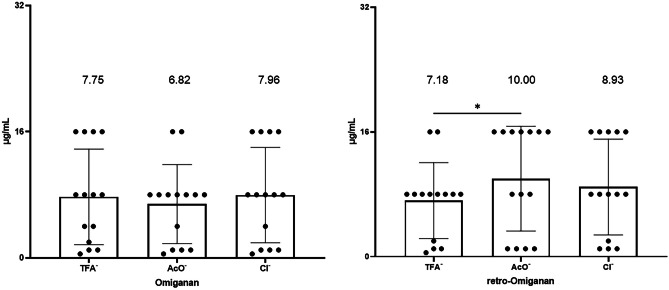
Minimum inhibitory concentrations of omiganan and retro-omiganan against reference and clinical strains of staphylococci [µg/mL]. The figures represent the mean of at least 3 biological replicates ± SD (*p < 0.05 was considered significant)

Antimicrobial activity studies were carried out against reference strains (5) from the ATCC collection and clinical strains (9), isolated from patients with atopic dermatitis. The strains were selected for testing based on previous MIC analyses, based on which those characterized by the highest resistance to conventional antibiotics (MIC values against conventional antibiotics are presented in supplementary Table S1.) [29]. The MIC results indicate that all compounds inhibited the growth of staphylococci over the concentration range of 0.5 to 16 μg/mL. The most active compounds were omiganan acetate and retro-omiganan trifluoroacetate, for which the average MIC values were 6.82 and 7.18 µg/mL, respectively (Fig. 4). Interestingly, only a significant change in inhibitory characteristics was noticed in retro-omiganan acetate, whose overall MIC values were higher compared to those of the initial compound (TFA^−^).

**Fig. 5:**
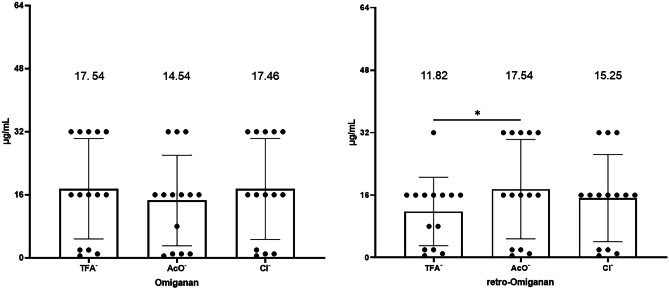
Minimum biofilm inhibitory concentrations of omiganan and retro-omiganan against reference and clinical strains of staphylococci [µg/mL]. The figures represent the mean of at least 3 biological replicates ± SD (*p < 0.05 was considered significant)

Another parameter that was investigated in microbiological assays was the ability to inhibit biofilm formation on the polystyrene surface of 96-well plates. In this case, the highest potential was found for retro-omiganan trifluoroacetate, for which the average MBIC value was 11.82 μg/mL, and the scatter of results for all compounds oscillated between 0.5 and 32 μg/mL (Fig. 5). Interestingly, also this case, a significant change in inhibitory properties against biofilm was found for retro-omiganan acetate, which again appeared to be the weakest compound.

**Fig. 6:**
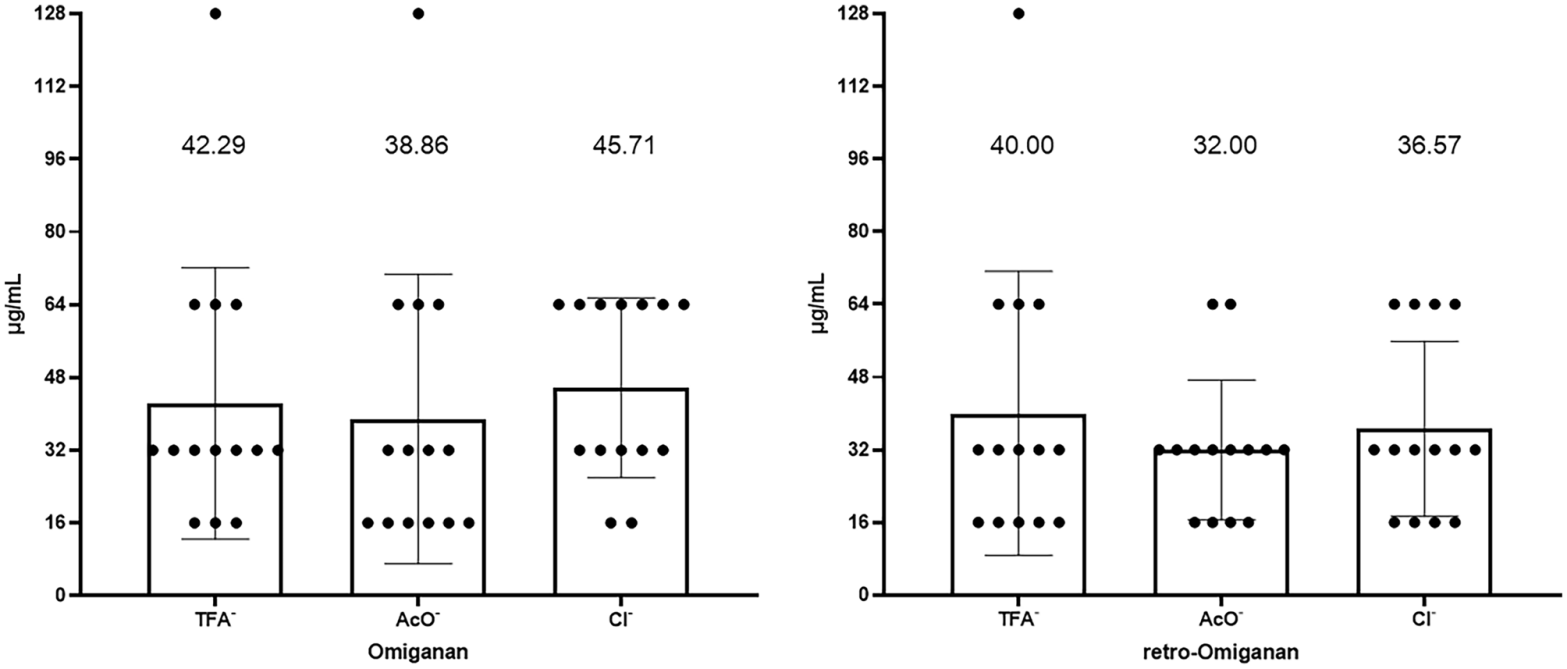
Minimum biofilm eradication concentrations of omiganan and retro-omiganan against reference and clinical strains of staphylococci [µg/mL]. The figures represent the mean of at least 3 biological replicates ± SD three (*p < 0.05 was considered significant)

Eradication of the already formed, 24-h biofilm for the tested AMPs was noticed at almost twofold and fourfold higher concentrations as compared to those of MIC and MBIC values, but it was characterized the by definitely narrowest range of activity. The lowest MBEC concentrations were 16 μg/mL while the highest one was 128 μg/mL (Fig. 6). It should be noted that those highest values were found only for one reference strain (*S. aureus* ATCC 25923). Interestingly, the lowest mean MBEC was determined for retro-omiganan acetate (32 μg/mL). This finding is in contrast to the previous assays in which it appeared to be the least potent compound. Moreover, in general, acetates exhibited the highest activity against staphylococcal biofilm, but the change in the activity was insignificant. All experiments were conducted at least three-independent experiments. Exact values for each strain are shown in supplementary Tables S2-S4. Moreover, all the obtained results suggest that the lowest MIC value was noticed for omiganan acetate, MBIC for retro-omiganan trifluoroacetate, while MBEC for retro-omiganan acetate. Furthermore, based on the results of analyses of the peptide content, the adjusted mean of the MIC, MBIC, and MBEC concentrations was determined (Table 4). Despite clear differences in the effective concentration of the peptides after counterion exchange, the lowest MIC value was still exhibited by omiganan acetate, while for MBIC it was retro-omiganan trifluoroacetate, and eradication of biofilm was found to be the most effective with retro-omiganan acetate. Based on these analyses, it can be claimed that the most favorable antistaphylococcal properties for both compounds are determined by the presence of the acetate counterion, and the highest activity against biofilm shows retro-omiganan.

**Table 4:** The results of microbiological assays after consideration of peptide content after counterion exchange in terms of mean values of MIC, MBIC, and MBEC [µg/mL]

**Peptide**	**Counterion**	**Initial values**			Corrected values		
		**MIC**	**MBIC**	**MBEC**	**MIC**	**MBIC**	**MBEC**
Omiganan	TFA^−^	7.75	17.54	42.29	7.75	17.54	42.29
Retro-omiganan		7.18	11.82	40.00	6.10	10.05	34.00
Omiganan	AcO^−^	6.82	14.54	38.86	5.39	11.48	30.70
Retro-omiganan		10.00	17.54	32.00	7.50	13.15	24.00
Omiganan	Cl^−^	7.96	17.46	45.71	6.77	14.84	38.86
Retro-omiganan		8.93	15.25	36.57	8.57	14.64	35.11

### MTT Assay

The results of measurements of cytotoxicity to the human keratinocyte cell line (HaCaT) indicate that the least toxic appeared to be omiganan trifluoroacetate, with the IC_50_ value of 77.10 μg/mL (Table 5). However, considering the average MIC values against the tested strains of *S. aureus*, the highest selectivity index (SI) was found for omiganan acetate (10.81). In addition, these analyses indicate that sequence reversal results in up to a threefold increase in cytotoxicity. Consequently, retro-omiganan acetate was found to be the most cytotoxic against HaCaT cell line, with IC_50_ of 23.66 μg/mL and a selectivity index of 2.37.

**Table 5:** The IC_50_ values of compounds with different counterions and calculated selectivity indexes

	**Counterion**	**IC**_**50**_	**MIC**	**SI**
Omiganan	**TFA**^**−**^	77.10	7.75	9.94
Retro-omiganan	**TFA**^**−**^	29.51	7.18	4.11
Omiganan	**AcO**^**−**^	73.72	6.82	10.81
Retro-omiganan	**AcO**^**−**^	23.66	10.00	2.37
Omiganan	**Cl**^**−**^	63.99	7.96	8.04
Retro-omiganan	**Cl**^**−**^	26.66	8.93	2.99

### Hemolytic Activity

**Fig. 7:**
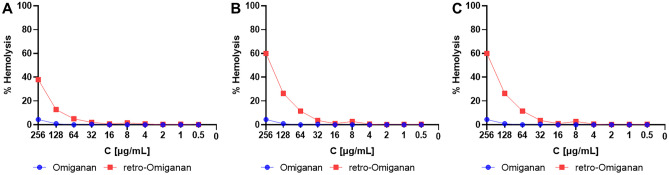
Hemolytic activity of the peptides with different counterions. **A** Trifluoroacetates; **B** acetates; **C** chlorides

Among all tested salts, retro-omiganan appeared to be more hemolytic than omiganan (Fig. 7). Interestingly, only omiganan trifluoroacetate did not cause significant erythrocyte hemolysis (> 10%) over the entire range of the concentrations (Fig. 1A). With the remaining compounds, higher values were found, and it appeared that the exchange of counterions resulted in more hemolytic compounds. The highest degree of hemolysis was noted for retro-omiganan chloride and acetate (60.12 and 59.99%, respectively) at a concentration of 256 μg/mL. Moreover, the lowest concentration of these compounds that caused hemolysis > 10% (16.02 and 11.56%) was recorded at a concentration of 64 μg/mL.

### Membrane Depolarization

**Fig. 8:**
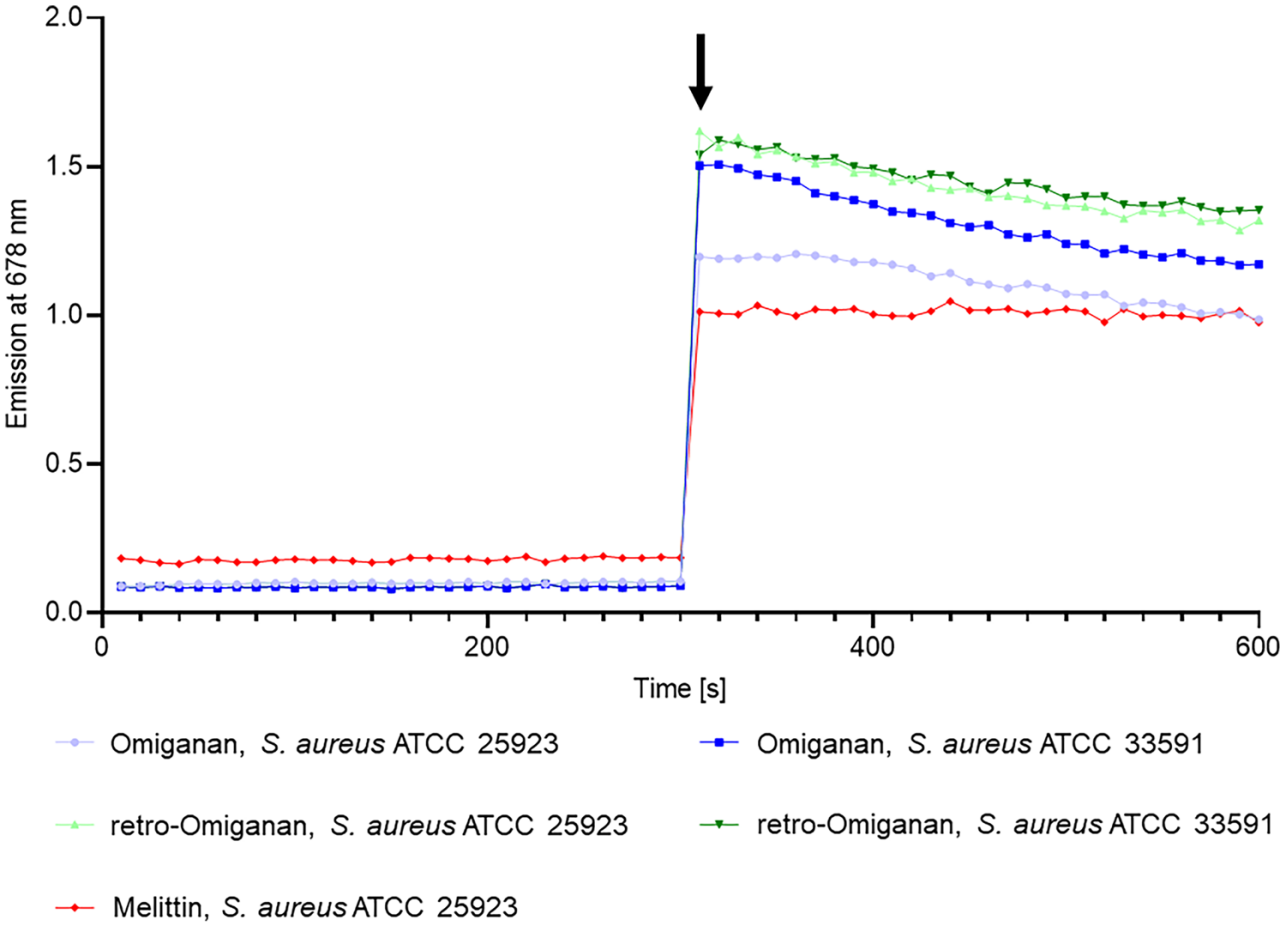
Results of fluorescence measurements of the membrane potential-sensitive probe

Analyses of membrane depolarization were performed using the reference strains of *S. aureus* ATCC 25923 and *S. aureus* ATCC 33591 (MRSA). The control compound was melittin as it is a model membrane-disrupting peptide [43]. The results of fluorometric analyses indicate that omiganan and retro-omiganan cause depolarization of *S. aureus* membranes immediately after the addition of the compounds at a concentration of 2 × MIC. This is accompanied by an immediate increase in fluorescence that persists during the analysis (Fig. 8), which proves the membrane depolarization potential of omiganan and retro-omiganan.

### Impact of Exposition Under Flow on Susceptibility Profile of S. aureus Reference Strains

In these experiments, the impact on MIC values of pre-treatment (24 to 72 h) of the biofilm formed under flow conditions with omiganan and its retro-analog was investigated. As a result, it was shown that generally it did not significantly affect the sensitivity of selected strains of *S. aureus* to the majority of conventional antibiotics. However, it was observed that the sensitivity of *S. aureus* strain ATCC 25923 to ciprofloxacin and omiganan decreased (½ baseline MIC) after both omiganan and its retro-analog treatment just after 24 h of biofilm treatment (Table S5). A twofold decrease in MIC concentrations for daptomycin was also noted for this strain after treatment with retro-omiganan. It is also worth mentioning that pre-treatment with omiganan and retro-omiganan resulted in a stable ½ baseline MIC of omiganan itself. In the case of the MRSA reference strain (*S. aureus* ATCC 33591), pre-treatment with AMPs affected the susceptibility to daptomycin and the peptide themselves. In the case of the first one, the lower MIC values (1 µg/mL as compared to the initial 2 µg/mL) were noticed for those biofilm-associated bacteria that were treated for at least 48 h with retro-omiganan. It is noteworthy that the bacteria exhibited twofold increase in sensitivity to those peptides after pre-treatment, with initial values of 16 µg/mL decreasing to final values of 8 µg/mL (Table S6).

### Impact of Co-immobilized Peptides on Biofilm Under Flow

**Fig. 9:**
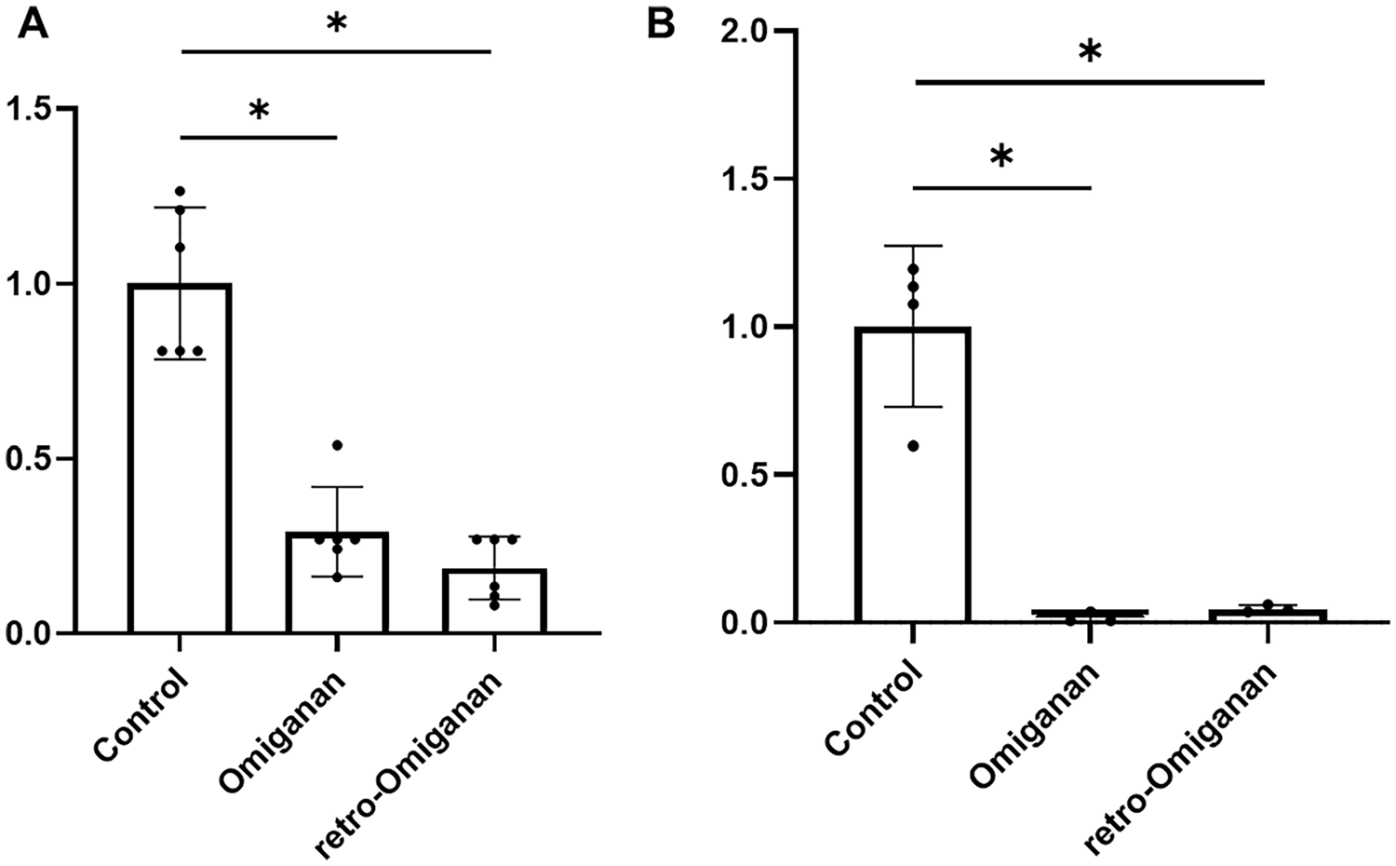
Antimicrobial activity of coated PDMS channels against **A** *S. aureus* ATCC 25923 and **B** *S. aureus* ATCC 33591. The reduction ratio (%) relative to the control. The figures represent the mean of 3 biological replicates (3 technical replicates each) ± SD of three (*p < 0.05 was considered significant)

As part of the studies in the flow model, the effect of incorporation of omiganan and retro-omiganan into the inner side of the PDMS channels was examined to assess how these compounds could affect the formation of biofilm. The amount of bound peptides (internal area of ca.100 mm^2^) was estimated by RP-HPLC analyses of the solution used for functionalization, before and after the experiment. For omiganan, it was 12.48 ± 0.22 µg while for retro-omiganan it was 12.35 ± 0.10 µg. After the introduction of the peptides to the channels, the microbiological assays followed. Interestingly, it appeared that the number of bacteria that were able to attach and form biofilm in functionalized channels was significantly lower for both reference *S. aureus* strains and both compounds as well. The highest degree of reduction of 98 and 96% showed MRSA reference strain (*S. aureus* ATCC 33591) as compared to that of control for omiganan and retro-omiganan, respectively (Fig. 9B). For *S. aureus* ATCC 25923, a lower degree of reduction was noticed, but still higher for retro-omiganan (81%) than that of omiganan (71%) (Fig. 9A).

## Discussion

To date, many approaches have been utilized to design and obtain AMPs with improved antimicrobial properties, such as cyclization, dimerization, deletion and substitution of amino acids, glycosylation, and lipidation [44–48]. Here, we focused on two main approaches that were exploited in our lab, namely, synthesis of retro-analogs and the exchange of counterions. To answer how these changes could influence the antimicrobial properties, comprehensive microbiological studies on *S. aureus* reference and clinical strains were conducted. In our previous study, among several AMPs, a retro-analog of omiganan (retro-omiganan) was found to be characterized by enhanced antistaphylococcal activity [22]. Moreover, since omiganan is one of the most extensively studied AMPs in clinical trials, it was reasonable to focus on this particular compound. Nevertheless, many other studies indicate that this approach results in AMPs with improved antimicrobial activity. For instance, Subbalakshmi et al. in studies on the activity of synthetic analogs of SPF peptide (the most hydrophobic fragment of bovine seminalplasmin) proved that the compounds with reversed sequence showed comparable or even higher activity against *Escherichia coli*, *Pseudomonas aeruginosa*, and *S. aureus* [49]*.* It is worth mentioning that, despite an increase in bactericidal properties, hemolytic activity of these compounds remained at the same level. Similar relationships were reported by Gopal et al., who compared the activity of 6-amino acid peptide (WK)3. Moreover, a retro-analog showed twice as high activity against all the tested microorganisms (gram-positive and gram-negative bacteria and fungi) but did not affect hemolytic activity [50]. In this study, reversion of the sequence did not affect staphylococcal activity in terms of MIC values. Interestingly, a striking difference upon comparing the antimicrobial activity was noticed for biofilm inhibition (MBIC values), and its eradication was also more efficient for retro-analog. Moreover, in contrast to the previously mentioned research, the reversion of the sequence leads to more hemolytic compounds; however, microbiologically effective concentrations (also those against biofilm) were much lower than those responsible for the significant degree of hemolysis of human erythrocytes. In fact, Faccone et al. classified omiganan as one of the AMPs with a low hemolytic potential [51]. Of course, our studies did not concentrate only on the comparison of reversion of the sequence. Here, we strive to focus on the importance of the counterion component in terms of antimicrobial activity. Several researchers highlight the toxicity of TFA, including inhibition of cell proliferation and need for exchange to biocompatible ones [52]. Moreover, the disruption of target cell membranes of microorganisms by AMPs depends on the peptide characteristics such as well-defined hydrophobic and hydrophilic faces which are associated with specific counterions. Our previous studies of counterion on influence on the activity of AMPs and other researchers confirm the necessity for testing different salt forms of peptides [53–55]. Furthermore, currently available peptide-based drugs are generally hydrochlorides and acetates [56], thus AMPs for this study were obtained as trifluoroacetates (TFA^−^), acetates (AcO^−^), and chlorides (Cl^−^). The results of antimicrobial assays indicate that the acetate is a profitable form of salt for both peptides (lowest MIC and MBEC). On the other hand, retro-omiganan trifluoroacetate turned out to be an excellent compound for biofilm inhibition. Similarly, the most beneficial ratio of antimicrobial activity and cytotoxicity was obtained for omiganan acetate and trifluoroacetate, and the least one for chloride. These results are compatible with other ones carried out on this compound. For instance, Zapotoczna et al. [57] reported that the MIC values for d-omiganan (all amino acids with d-configuration) and MRSA strains were 8 µg/mL. Furthermore, the MBEC values for different strains after 6 h of treatment ranged from 18.6 to 20.8 µg/mL. On the other hand, in study of Ng et al. [58], omiganan and d-omiganan were compared in terms of activity against mupirocin-resistant MRSA strains. For both compounds and all strains analyzed, the MIC values were equal to 12.5 µM (22.7 µg/mL). It is worth mentioning that the use of d-amino acids in the sequence increases the resistance of peptides to proteases, which translates positively into their potential application. In the previously mentioned work, after 1 h of incubation with human skin S9 protease, omiganan appeared to be unstable with a ca. 10 min half-life, while d-omiganan remained stable for over 2 h at 80% of its amount. It should be noted that the research on omiganan is mostly focused on its chloride salts. Interestingly, omiganan pentahydrochloride has been subjected to the majority of clinical trials [59–61] along with its involvement in other research on this compound. In the work of Sader et al. [62], studied omiganan pentachloride on a large number of clinical strains, including oxacillin-sensitive *S. aureus* and oxacillin-resistant MRSA strains. In both groups, the MIC values ranged between 2 and 64 µg/mL. Furthermore, Fritsche et al. reported MIC_50_ and MIC_90_ values for omiganan pentachloride against MSSA, MRSA, VISA, and VRSA clinical strains (*n* = 109) ranging between 16 and 32 µg/mL [63]. However, in the light of the present results, omiganan should be reconsidered and studied using another biocompatible form of salt, e.g., acetate. For instance, in our previous studies on the antistaphylococcal activity of different salts, MIC values of acetates were lowest for pexiganan and temporin A [23]. A frequently verified parameter in research on new AMPs, assessment of membrane depolarization using DiSC_3_(5) has been included [64–66]. The DiSC3(5) is a cationic membrane potential-dependent dye. In the absence of membrane-depolarizing agent, dye is accumulated on hyperpolarized membranes and is translocated into the lipid bilayer. This location of the probe results in quenching of the fluorescence. Peptides can cause membrane depolarization, and as a result the probe is released to the medium and increased fluorescence can be observed. Here, the analyses were carried out using reference strains: *S. aureus* ATCC 25923 and *S. aureus* ATCC 33591 (MRSA). The results indicate that omiganan and retro-omiganan cause depolarization of bacterial membranes immediately after their addition at concentrations of 2 × MIC. This is accompanied by an immediate increase in fluorescence throughout the duration of the analysis. It can be deduced that one of the mechanisms underlying the antimicrobial activity of omiganan and its retro-analog includes depolarization of the cell membrane. Although such a study with omiganan has not been reported so far, the potential for membrane depolarization was determined. For example, Fritsche et al. determined interactions of this peptide in a laboratory model of cell membranes and walls [63]. The authors based on a number of mathematical analyses and modeling supported by biological studies showed a relationship between MIC concentration and interaction with *S. aureus* membranes. In addition, a high affinity between the peptide and peptidoglycan was claimed. On the other hand, Friedrich et al. [67] in studies on the activity of hybrid peptides CP26, CP29, and analog of indolicidin, CP11CN, also assessed the degree of membrane depolarization [64]. Interestingly, these studies showed that CP26 the lowest antimicrobial activity showed an excellent ability to permeabilize membranes. Again, CP11CN and indolicidin, despite the fact that they led to membrane depolarization at low concentrations, caused this in up to 75% of cells. Another aspect investigated in this paper was measurement of the impact of exposition under flow on the susceptibility profile of *S. aureus* strains. To do this, two representative reference strains were used, namely, *S. aureus* ATCC 25923 and *S. aureus* ATCC 33591 (MRSA). Moreover, to simulate the continuously varying conditions, a flow model of investigation against biofilm was applied. As a result, it appeared that staphylococcal biofilms treated with omiganan became more sensitive to it. Identical behavior was noticed for ciprofloxacin. In the case of MRSA strains, a decrease in susceptibility to daptomycin was also observed only after 48 h of treatment with retro-omiganan. Interestingly, treatment of biofilm with retro-omiganan induced a reciprocal, twofold decrease in MIC concentrations in both strains. It is worth noting that the treatment of these strains using both compounds did not affect the susceptibility profile for other antibiotics tested. To date, no research has been undertaken to investigate the effects of biofilm exposure to different antimicrobial compounds under flow and a further impact on the sensitivity profile of bacteria associated with this biofilm. In fact, most of these studies are based on serial passages followed by an assessment of antibiotic susceptibility [68, 69]. However, bacteria are mostly exposed to a number of AMPs in their environment, in particular those involved in the immune reactions of infected organisms. In addition, resistance to AMPs can be either passive or induced during the infection. For example, congenital resistance to AMPs occurs in bacteria of *Burkholderia* spp., *Morganella* spp., *Proteus* spp., *Providencia* spp., and *Serratia* spp., which are characterized by more positively charged molecules of lipid A (a component of LPS), which reduces interactions with AMPs [70]. For *S. aureus*, there are a number of mechanisms of resistance to AMPs including LL-37, human defensins, or lactoferricin B. These mechanisms include, among others, the ability to produce a number of proteolytic enzymes, the presence of regulatory systems that along with AMPs affect the expression of membrane proteins that specifically bind to peptide molecules, innate resistance of SCV (small colony variants), and finally the formation of biofilm [71, 72]. Importantly, biofilm-associated bacteria exposed to AMPs have a higher propensity to develop resistance to these compounds and consequently lead to the occurrence of cross-resistance, e.g., to endogenous peptides [73]. For this reason, it seems worthwhile to carry out resistance induction studies within a flow-based research model that reflects conditions encountered in vivo since bacteria are constantly exposed to stable concentrations of test compounds. Referring to the results of this study, it is expedient to highlight suppresses in sensitivity to daptomycin as an unprecedented phenomenon. Mainly due to the fact that more and more attention has been paid to the fact that cross-resistance to daptomycin and endogenous antimicrobial peptides occurs in *S. aureus* [27, 74, 75]. On the other hand, some data suggest that staphylococci exposed to a mixture of AMPs show a weaker tendency for development resistance to AMPs [76]. Undoubtedly, the key aspect of the fight against infections caused by multi-resistant strains of microorganisms, including *S. aureus*, is prevention. With reference to the guidelines of the European Society of Clinical Microbiology and Infectious Diseases (ESCMID), prevention and education are the most important aspects for effectively combating nosocomial infections [77]. This includes an appropriate antibiotic policy, adequate infrastructure, and procedures to prevent the spread of pathogens. However, in addition to existing health services, the achievements of research groups aimed at the development of new antimicrobial substances and their methods of application are also important [78–80]. Therefore, the combination of these two aspects can minimize the spread of infections caused by multi-resistant microbes and prevent the development of resistance. The incorporation of antibacterial compounds into biomaterials prevents colonization and biofilm formation. The flow model study on the effect of incorporation of omiganan and retro-omiganan into the PDMS channels is helpful in assessing how these compounds might influence biofilm formation of *S. aureus* strains. As previously mentioned, among the tested salts and retro-analogs, the retro-omiganan trifluoroacetate appeared to be prominent for functionalization of the surfaces, thus making it a favorable compound for biomaterial preparation. Nevertheless, it would be reasonable to conduct such studies using different pathogenic species, multi-species biofilm, or even other kinds of fluids to mimic more precisely conditions encountered in real-world scenarios. Also, the stability of peptide-pDa-PDMS binding would be reasonable for the potential application of such an approach. Despite the fact that the innovative aspect of the current study, other AMPs were used in studies on biomaterials. According to our knowledge, research into the antistaphylococcal activity of biomaterials coated with omiganan has never been conducted to date. It was shown that besides antimicrobial activity it has also immunomodulatory potential and in combination with imipenem can improve mucosal re-epithelialization [81, 82]. Moreover, some AMPs structurally similar to omiganan were used as antibiofilm coatings, e.g., peptide E6 (arginine-rich—RRWRIVVIRVRRC) and TetraF2W-RR (arginine- and tryptophan-rich—WWWLRRIW) [83]. Another example is CysTetraF2W-RR, for which a coated polyethylene terephthalate (PET) surface turned out to significantly reduce a fraction of *S. aureus* cells [84]. In other research works, similar aspects to those explored in this study have also been investigated. For instance, Klermund et al. [85] conducted research on the incorporation of 3 “peptide anchors” showing no antimicrobial activity to evaluate functionalization strategies. For these compounds, the number of bound peptides into the PDMS surface was 6, 10, and 26% being compatible with those of our research [81]. In turn, in the work of Lim et al. [86] on PDMS binding of the peptide CWR11 (CWFWKWWRRRRRR-NH_2_) using polydopamine was evaluated by verifying surface morphology using atomic force microscopy and spectroscopic detection of amide bonds (ATR-FTIR) [82]. Although these determinations were rather qualitative, the key aspect was the assessment of bacterial colony count after 3 h of incubation in bacterial suspensions of *E. coli*, *P. aeruginosa*, and *S. aureus*. As a result, for functionalized PDMS fragments with CWR11 peptide, no increase in bacterial colonies was observed as compared to that of the control. The disadvantage of this approach was that the idea of the work was to further use CWR11 peptide for functionalization of intravascular catheters, and microbiological tests were carried out in stationary conditions. Considering the use of polydopamine and PDMS, it can be presumed that omiganan is covalently bonded to polydopamine. However, some of the peptide molecules can be released after surface functionalization. Peptide release has previously been observed with polydopamine-KR-12 coated titanium alloys and therefore it can also be expected for omiganan and retro-omiganan [87]. This might be a result of possible co-occurrence of non-covalent interactions between AMPs and polydopamine, e.g., π–π electron interaction (tryptophan and dopamine) or hydrogen bonds [88]. Hypothetically, omiganan and retro-omiganan anchored to a surface could reduce cell adhesion and disrupt cells through the contact-kill mechanism. Owing to the positive charge of these compounds, it can be speculated that negatively charged bacteria cells would be attracted by the PDMS surface, but the amphiphilic nature of these molecules and their established membrane-disruptive ability would lead to cell death [62, 89, 90]. This study supports the thesis that omiganan as well as its retro-analog can be used to modify surfaces to prevent *S. aureus* colonization, especially in the case of catheter-associated urinary tract infections (CAUTIs) where antimicrobial peptides can be used for antimicrobial catheters. It is worth mentioning that despite the implementation of hygiene procedures, CAUTIs remain common in hospital settings [91]. However, antibiotics or silver coatings have become increasingly used for addressing this issue, although they are characterized by several disadvantages. For instance, a major concern for antibiotics is their contribution to antibiotic resistance development [92, 93]. On the other hand, silver loses its antimicrobial activity over extended periods, can be cytotoxic, and may not always be suitable for long-term catheterization [94]. For this purpose, omiganan or retro-omiganan for their wide-spectrum of activity could be evaluated. While our investigation suggests that exchange of counterion could be justifiable for enhancing bacteria eradication, its significance concerning the prevention of bacterial colonization and biofilm formation is lower and not so essential. Besides these findings, we also present a unique flow-based model for studying biofilms which contrary to microfluidics, lab-on-a-chip approaches used for studying biofilm can be inexpensively replicated. Although this model was applied for studies on *S. aureus* strains, so it is hard to estimate how other bacterial species would behave, especially those bacteria, which are characterized by slime production such as *P. aeruginosa* or *Klebsiella pneumoniae*. Nevertheless, the easiness of our approach allows to modify the channel length and diameter also can be used for live monitoring devices.

## Conclusions

In this study, we have pointed out the importance of counterions in designing of AMPs and their further antimicrobial activity [56]. It can be stated that retro-omiganan TFA^−^ had the highest biofilm inhibitory properties while AcO^−^ of omiganan and its retro-analog were the most efficient against planktonic and biofilm cultures. At the same time omiganan AcO^−^ had the greatest selectivity index. Concluding, counterion can affect peptide biological activity; however, different effects can be expected between salts. In the light of the above, it is reasonable to consider counterion exchange in optimization of peptide therapeutic potential. In addition, in both compounds (omiganan and retro-omiganan), there was no tendency to develop resistance among the tested strains, hence their high application potential. An even more significant conclusion is that we demonstrated the high effectiveness of omiganan and retro-omiganan in preventing bacterial adhesion. Bearing in mind that the effective amount of a peptide bound to the surface of a polydopamine functionalized PDMS channel is many times lower than its cytotoxic concentration, the activity not only against *S. aureus*, but also against other microorganisms appears to be decisive. In the case of retro-omiganan, an increased activity was found previously against a number of microorganisms, including *E. coli*, *P. aeruginosa*, *K. pneumoniae*, as well as *Candida albicans*. The enhanced activity of retro-omiganan against a wide range of microorganisms suggests that it may likely exhibit even a higher application potential than omiganan. However, further extensive exploration should be continued. The developed model of studying biofilms under flow conditions seems to be a simple and adequate tool for further research.

## Supplementary Information

Below is the link to the electronic supplementary material.

Supplementary file1 (PDF 106 KB)

Supplementary file2 (PDF 65 KB)

Supplementary file3 (PDF 66 KB)

Supplementary file4 (PDF 65 KB)

Supplementary file5 (PDF 93 KB)

Supplementary file6 (PDF 93 KB)

## Data Availability

All research data and strains used in this study are available from corresponding authors with reasonable request.
