# Supplementary material for: The Study of Antistaphylococcal Potential of Omiganan and Retro-Omiganan Under Flow Conditions
Source: Probiotics Antimicrob Proteins. 2024 Jan 15;17(3):1447–65. doi: 10.1007/s12602-023-10197-w (PMC12055641; doi:10.1007/s12602-023-10197-w)
Supplement: Supplementary file 1 — Supplementary file1 (PDF 106 KB) [file 12602_2023_10197_MOESM1_ESM.pdf]

Table S1. Antimicrobial susceptibility (MIC) of clinical isolates used in the study [µg/mL].

Strain	Ampicillin	Ciprofloxacin	Daptomycin	Erythromycin	Fusidic Acid	Linezolid	Lincomycin	Mupirocin	Tetracycline	Vancomycin
004N (MRSA)	0.5	0.25	2	256	0.0625	1	1	0.125	0.5	0.5
005S	8	0.125	4	0.125	0.0625	0.5	1	>512	0.5	0.125
009S	0.125	2	0.5	0.125	2	0.25	0.25	>512	1	0.25
015N (MRSA)	16	0.25	2	32	0.125	2	1	0.25	0.5	1
030N	0.5	2	0.125	0.25	0.25	0.5	0.5	0.25	0.5	2
031S	0.5	2	0.125	0.125	0.25	2	0.5	32	0.25	0.25
043SC (MRSA)	32	0.25	1	>512	0.125	2	>512	0.5	32	0.5
051N (MRSA)	0.25	0.25	2	0.125	0.125	2	1	0.25	0.25	0.5
060S	0.25	1	0.125	0.125	0.125	1	0.5	>512	0.125	0.5

N – strain was isolated from nasal swab
S – strain was isolated from skin
SC – strain was isolated from skin of patient with furuncle folliculitis
▢ – strain was resistant to test antibiotic (based on clinical breakpoints data of EUCAST - https://www.eucast.org/clinical_breakpoints)
