# Supplementary material for: The Study of Antistaphylococcal Potential of Omiganan and Retro-Omiganan Under Flow Conditions
Source: Probiotics Antimicrob Proteins. 2024 Jan 15;17(3):1447–65. doi: 10.1007/s12602-023-10197-w (PMC12055641; doi:10.1007/s12602-023-10197-w)
Supplement: Supplementary file 2 — Supplementary file2 (PDF 65 KB) [file 12602_2023_10197_MOESM2_ESM.pdf]

**Table S2.** Minimal Inhibitory Concentrations of Omiganan and retro-Omiganan against reference and clinical strains of staphylococci [µg/mL].

| | Omiganan | | | retro-Omiganan | | |
|---|---|---|---|---|---|---|
| | **$TFA^-$** | **$AcO^-$** | **$Cl^-$** | **$TFA^-$** | **$AcO^-$** | **$Cl^-$** |
| ATCC 25923[1] | 16 | 16 | 16 | 8 | 16 | 16 |
| ATCC 33591 (MRSA) [1] | 16 | 16 | 16 | 16 | 16 | 16 |
| ATCC 6538[1] | 4 | 8 | 8 | 8 | 8 | 8 |
| ATCC 9144[1] | 4 | 4 | 4 | 8 | 8 | 8 |
| ATCC 12598[1] | 8 | 8 | 8 | 8 | 8 | 16 |
| 004N (MRSA) [2] | 8 | 8 | 8 | 8 | 16 | 16 |
| 005S[2] | 1 | 1 | 1 | 0.5 | 1 | 1 |
| 009S[2] | 0.5 | 0.5 | 0.5 | 1 | 1 | 1 |
| 015N (MRSA) [2] | 16 | 8 | 16 | 8 | 16 | 8 |
| 030N[2] | 8 | 8 | 16 | 16 | 16 | 8 |
| 031S[2] | 1 | 1 | 1 | 2 | 1 | 2 |
| 043SC (MRSA) [2] | 8 | 8 | 8 | 8 | 16 | 8 |
| 051N (MRSA) [2] | 16 | 8 | 8 | 8 | 16 | 16 |
| 060S[2] | 2 | 1 | 1 | 1 | 1 | 1 |

[1] – reference strain
[2] – clinical strain
