# Supplementary material for: The Study of Antistaphylococcal Potential of Omiganan and Retro-Omiganan Under Flow Conditions
Source: Probiotics Antimicrob Proteins. 2024 Jan 15;17(3):1447–65. doi: 10.1007/s12602-023-10197-w (PMC12055641; doi:10.1007/s12602-023-10197-w)
Supplement: Supplementary file 4 — Supplementary file4 (PDF 65 KB) [file 12602_2023_10197_MOESM4_ESM.pdf]

Table S4. Minimal Biofilm Eradication Concentrations of Omiganan and retro-Omiganan against reference and clinical strains of staphylococci [µg/mL].

	**Omiganan**			**retro-Omiganan**		
	**TFA^-**	**AcO^-**	**Cl^-**	**TFA^-**	**AcO^-**	**Cl^-**
ATCC 25923[1]	128	128	64	128	64	64
ATCC 33591 (MRSA) [1]	32	64	64	64	32	32
ATCC 6538[1]	32	64	32	64	32	64
ATCC 9144[1]	32	16	32	32	32	32
ATCC 12598[1]	32	32	64	32	32	32
004N (MRSA) [2]	64	32	64	64	32	64
005S[2]	16	16	32	16	16	16
009S[2]	32	16	32	16	16	16
015N (MRSA) [2]	64	32	64	32	32	32
030N[2]	32	32	64	32	32	32
031S[2]	16	16	16	16	16	16
043SC (MRSA) [2]	32	16	32	16	32	32
051N (MRSA) [2]	64	64	64	32	64	64
060S[2]	16	16	16	16	16	16

[1] – reference strain
[2] – clinical strain
