# Supplementary material for: The Study of Antistaphylococcal Potential of Omiganan and Retro-Omiganan Under Flow Conditions
Source: Probiotics Antimicrob Proteins. 2024 Jan 15;17(3):1447–65. doi: 10.1007/s12602-023-10197-w (PMC12055641; doi:10.1007/s12602-023-10197-w)
Supplement: Supplementary file 5 — Supplementary file5 (PDF 93 KB) [file 12602_2023_10197_MOESM5_ESM.pdf]

Table S5. MIC values [μg/mL] of Omiganan and retro-Omiganan-treated *S. aureus* 25923

		Omiganan			Retro-Omiganan		
**Time [h]**	0	24	48	72	24	48	72
Ampicillin	0.5	0.5	0.5	0.5	0.5	0.5	0.5
Ciprofloxacin	1.0	**0.5**	**0.5**	**0.5**	**0.5**	**0.5**	**0.5**
Daptomycin	2.0	2.0	2.0	2.0	**1.0**	**1.0**	**1.0**
Erythromycin	⩽ 0.25	⩽ 0.25	⩽ 0.25	⩽ 0.25	⩽ 0.25	⩽ 0.25	⩽ 0.25
Fusidic Acid	⩽ 0.25	⩽ 0.25	⩽ 0.25	⩽ 0.25	⩽ 0.25	⩽ 0.25	⩽ 0.25
Linezolid	1.0	1.0	1.0	1.0	1.0	1.0	1.0
Lincomycin	1.0	1.0	1.0	1.0	1.0	1.0	1.0
Mupirocin	⩽ 0.25	⩽ 0.25	⩽ 0.25	⩽ 0.25	⩽ 0.25	⩽ 0.25	⩽ 0.25
Tetracycline	⩽ 0.25	⩽ 0.25	⩽ 0.25	⩽ 0.25	⩽ 0.25	⩽ 0.25	⩽ 0.25
Vancomycin	1.0	1.0	1.0	1.0	1.0	1.0	1.0
Omiganan	16.0	**8.0**	**8.0**	**8.0**	**8.0**	**8.0**	**8.0**
Retro-Omiganan	8.0	8.0	8.0	8.0	8.0	8.0	8.0
