# Supplementary material for: The Study of Antistaphylococcal Potential of Omiganan and Retro-Omiganan Under Flow Conditions
Source: Probiotics Antimicrob Proteins. 2024 Jan 15;17(3):1447–65. doi: 10.1007/s12602-023-10197-w (PMC12055641; doi:10.1007/s12602-023-10197-w)
Supplement: Supplementary file 6 — Supplementary file6 (PDF 93 KB) [file 12602_2023_10197_MOESM6_ESM.pdf]

Table S6. MIC values [µg/mL] of Omiganan and retro-Omiganan-treated *S. aureus* ATCC 33591 (MRSA)

		Omiganan			Retro-Omiganan		
**Time [h]**	0	24	48	72	24	48	72
Ampicillin	> 128	> 128	> 128	> 128	> 128	> 128	> 128
Ciprofloxacin	0.25	0.25	0.25	0.25	0.25	0.25	0.25
Daptomycin	2.0	2.0	2.0	2.0	2.0	**1.0**	**1.0**
Erythromycin	> 128	> 128	> 128	> 128	> 128	> 128	> 128
Fusidic Acid	⩽ 0.25	⩽ 0.25	⩽ 0.25	⩽ 0.25	⩽ 0.25	⩽ 0.25	⩽ 0.25
Linezolid	1.0	1.0	1.0	1.0	1.0	1.0	1.0
Lincomycin	> 128	> 128	> 128	> 128	> 128	> 128	> 128
Mupirocin	⩽ 0.25	⩽ 0.25	⩽ 0.25	⩽ 0.25	⩽ 0.25	⩽ 0.25	⩽ 0.25
Tetracycline	64.0	64.0	64.0	64.0	64.0	64.0	64.0
Vancomycin	1.0	1.0	1.0	1.0	1.0	1.0	1.0
Omiganan	16.0	**8.0**	**8.0**	**8.0**	**8.0**	**8.0**	**8.0**
Retro-Omiganan	16.0	**8.0**	**8.0**	**8.0**	**8.0**	**8.0**	**8.0**
